# Computed-Tomography-Structured Reporting in Middle Ear Opacification: Surgical Results and Clinical Considerations From a Large Retrospective Analysis

**DOI:** 10.3389/fneur.2021.615356

**Published:** 2021-02-24

**Authors:** Michele Cavaliere, Antonella Miriam Di Lullo, Camilla Russo, Massimo Mesolella, Elena Cantone, Giuseppe Di Lorenzo, Gaetano Motta, Andrea Elefante

**Affiliations:** ^1^Division of Otolaryngology-Head and Neck Surgery, Department of Neuroscience, Reproductive and Odontostomatologic Sciences, University of Naples “Federico II”, Naples, Italy; ^2^CEINGE-Centro di Ingegneria Genetica e Biotecnologie Avanzate, Naples, Italy; ^3^Neuroradiology Unit, Department of Advanced Biomedical Sciences, University of Naples “Federico II”, Naples, Italy; ^4^Head and Neck Surgery Unit, Department of Mental and Physical Health and Preventive Medicine, University of Campania “Luigi Vanvitelli”, Naples, Italy

**Keywords:** cholesteatoma, middle ear, computed tomography, surgical findings, chronic otitis media

## Abstract

**Purpose:** The aim of the study is to compare the accuracy of unstructured preoperative Computed Tomography (CT) reports from non-tertiary diagnostic centers with intraoperative findings in a large cohort of patients with Chronic Otitis Media (COM) undergone surgery.

**Methods:** From 2012 to 2019, a total number of 301 patients were considered for our purposes. All patients with clinical evidence of COM had preoperative non-contrast high resolution CT scan of the temporal bone in non-tertiary diagnostic centers, performed within 3 months before surgery.

**Results:** The accuracy of CT reports was analyzed in terms of nature, anatomical site, disease extension, bony erosion, vascular structures abnormalities relevant to surgical planning, and Eustachian tube patency. Compared to post-surgical findings, CT reporting critical analysis revealed a tendency to overestimation of bony erosion, coupled to underestimated description of facial canal/lateral semi-circular canal, vascular structures, and Eustachian tube.

**Conclusion:** Discrepancies between CT reports and surgical findings in middle ear opacification can be at least in part due to limited expertise of general radiologists in ENT neuroimaging. To limit this lack of information and the limited accuracy of middle ear structures depiction, here we propose a structured checklist to adopt in the case of a temporal bone CT scan for COM, in order to optimize the communication with surgeons and provide all the crucial information for an accurate surgical planning.

## Introduction

Chronic otitis media (COM) is a heterogeneous disorder with a complex pathogenesis, characterized by an altered tympanic membrane, occasional otorrhea, and conductive hearing loss (1). This disorder encompasses a wide spectrum of manifestations, ranging from simple COM to COM in the setting of specific diseases, from COM with granulation tissue to cholesteatoma (1). COM with cholesteatoma (and to a lesser extent COM with granulation tissue) can lead to the bony destruction of middle ear structures, generally involving the long process of the incus as more susceptible to erosion (1); when more pervasive, bony erosion can spread from the prominence of the horizontal semi-circular canals and the facial nerve canal to bony labyrinth, sigmoidal sinuses, and cranial cavities (1).

COM suspicion is raised at clinical examination (otoscopy, oto-endoscopy and micro-otoscopy), then supported by imaging techniques, such as computed tomography (CT) and magnetic resonance imaging (MRI) (2–5). Although CT is unable to define a proper assessment of lesion nature [so it is useful to integrate imaging detection with MRI diffusion weighted imaging (DWI)], it still remains the golden standard for evaluating the site of origin, the extent of disease and the possible involvement of crucial ear landmarks (e.g., facial canal, labyrinth, dural plate, tegmen tympani, scutum, ossicular chain) (3, 6–8). In particular, high resolution CT (HRCT) can provide crucial information to the surgeon, optimizing treatment planning (demolitive vs. conservative surgery), preventing complications and improving clinical outcome (8, 9). To this purpose, an accurate depiction of critical findings and an appropriate description of anatomical landmarks are strictly required in radiological reports. Indeed, it is undeniable that the accuracy of unstructured reports is generally influenced by radiologists' expertise in ear, nose, and throat (ENT) imaging; to overcome this possible inconvenience and limit inter-observer variability, structured reporting has been claimed as a model for improving quality and comparability of radiology reports (10).

With this background, the aim of this retrospective study was to compare preoperative CT reports with intra-operative features of patients' who have undergone surgery for COM, in order to evaluate the accuracy of non-tertiary diagnostic centers unstructured reports. We also propose a possible radiological checklist for middle ear pathology to couple with the radiological descriptive report, in order to facilitate the communication with clinicians and surgeons.

## Materials and Methods

A total number of 301 patients (57% males and 43% females; age range 18–78 years; mean age 48.3 years ±19.6) who underwent middle ear surgery at our university department between January 2013 and January 2020 were included in the retrospective study. The study was formerly approved by a local ethical committee; written informed consent was preliminarily obtained from all patients included in the study. Preoperative CT scans from more than 60 different non-tertiary diagnostic centers of patients suffering from COM (with or without cholesteatoma) were critically revised, with relative unstructured reports from more than 130 different non-ENT radiologists. CT images were further revised by two ENT radiologists (respectively, 5- and 15-years' experience) by using a structured reporting checklist (Supplementary Materials); at this stage, patients with post-traumatic tympanic perforation or confounding middle ear comorbidities were excluded from final analysis.

Pre-operative non-contrast HRCT scans of the temporal bone were performed within 1 month before surgery in different external non-tertiary diagnostic centers affiliated with the National Health System; all the examinations were performed on multi-detector scanners (minimum 16 slices) of different vendors, with the head in the neutral position. Non-contrast axial acquisitions were parallel to the major axis of the temporal bone (high-resolution matrix: 512 × 512; slice thickness: 0.6–1 mm; field of view: 15–20 cm); images were obtained with bone algorithm, and then reconstructed on multiple anatomic planes. The same ENT surgeon performed all surgical procedures at the Otorhinolaryngology Unit, University of Naples Federico II; a microscopic retro-auricolar approach was adopted in all cases, with final surgical revision of hidden spaces with endoscopic approach. Of all cases, 92 patients (31%) underwent a microscopic canal wall-up technique, 158 patients (52%) had a canal wall-down procedure, and 51 patients (17%) were treated by a radical mastoidectomy (Table 1). After surgery, for each patient the operator filled out a surgical report with data concerning lesion appearance, localization, and extension; the final diagnosis (simple COM and COM with granulation tissue, vs. COM with cholesteatoma) was then confirmed by pathological examination.

**Table 1:** Surgical procedures performed in our patients' cohort.

	**First look surgery**		**Second look surgery**		**Total**	
	***N***.	**%**	***N***.	**%**	***N***.	**%**
Tympanoplasty Canal wall up	72	24%	21	7%	93	31%
Tympanoplasty Canal wall down	133	44%	24	8%	157	52%
Radical mastoidectomy	51	17%	///	///	51	17%
Total	///	///	///	///	301	100%

**Figure 1:**
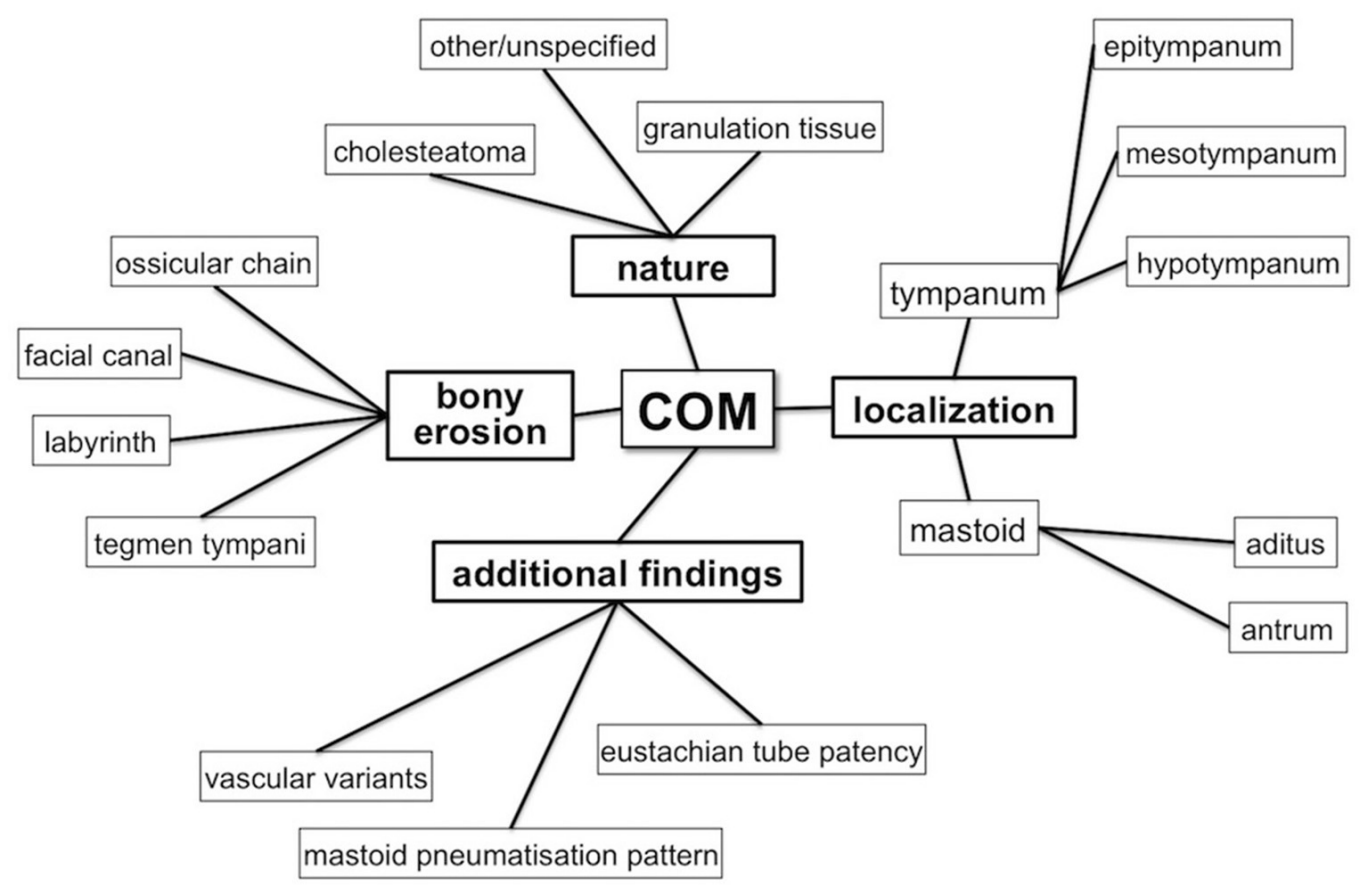
Diagram showing CT features considered for critical revision of unstructured reports.

All CT reports have been compared to surgical findings in terms of (Figure 1):

- lesion's nature (granulation tissue vs. cholestatoma);
- anatomical localization and disease extension (epitympanum, mesotympanum, hypotympanus, tympanic sinus, aditus ad antrum, antrum);
- erosion of ossicular chain (malleus, incus, stapes), tegmina tympani, tegmina antri, facial canal (tympanic tract, II knee and mastoid tract), labyrinthine structures [especially lateral semi-circular canal (LSC)];
- anatomical variants in vascular structures, both venous and arterial;
- patency state of the Eustachian tube.

Finally, inter-rater agreement was globally calculated by using Cohen's Kappa statistics (*k*) comparing surgical findings with unstructured CT reports, as well as surgical findings with structured CT checklist.

## Results

Differences between surgical findings and pre-operative surgical reports in our sample of 301 patients with COM are summarized in Table 2. On radiological reports, the lesion nature was hypothesized in 140 cases as cholesteatomas and in 106 cases as generically inflammatory, while in the remaining 54 cases no information concerning the possible nature of the lesion was provided; at surgery, the presence of cholesteatoma was documented in 195 cases (both isolated and admixed with granulation tissue), whereas granulation tissue only was found in 106 patients.

**Table 2:** Quantitative data analysis of intra-operative surgical findings vs. CT reporting vs. structured CT checklist in 301 patients with COM.

	**Intra-operative results**		**CT report**		**Structured CT checklist**	
	***N***	**%**	***N***	**%**	***N***	**%**
**Lesion nature**						
Cholesteatoma	195	62.9	140	46.5	169	56.1
Granulation tissue	106	37.1	106	35.2	132	43.9
Unspecified	0	0	54	18	0	0
**Lesion localization**						
Tympanic	301	100	132	43.8	301	100
Epitympanum	*287*	*95.3*	*72*	*23.9*	*285*	*94.7*
Mesotympanum	*78*	*25.9*	*33*	*10.9*	*75*	*24.9*
Hypotympanum	*91*	*30.2*	*15*	*30.2*	*80*	*26.6*
Mastoid	174	57.8	122	40.5	171	56.8
Aditus ad antrum	*174*	*57.8*	*14*	*4.6*	*171*	*56.8*
Antrum	*156*	*51.8*	*96*	*31.9*	*123*	*40.8*
**Ossicular chain erosion**	222	73.7	233	77.4	201	66.8
Malleus	*90*	*29.9*	*21*	*6.97*	*79*	*26.2*
Incus	*172*	*57.1*	*12*	*3.98*	*150*	*49.8*
Stapes	*131*	*43.5*	*0*	*0*	*64*	*21.3*
**Tegmen tympani**						
Erosion	20	6.64	60	19.9	35	11.6
Interruption	12	3.99	12	3.98	15	5.0
**Facial canal erosion**	76	25.2	18	5.98	51	16.9
Tympanic tract	*49*	*16.3*	*0*	*0*	*45*	*14.9*
Second knee	*23*	*7.64*	*0*	*0*	*15*	*5.0*
Mastoid tract	*4*	*1.33*	*0*	*0*	*0*	*0*
**Labyrinthine structures**						
Erosion	17	5.65	39	12.9	27	9.0
Incomplete fistula	13	4.32	9	2.99	17	5.6
Complete fistula	4	1.33	3	0.99	3	1.0
**Sigmoid sinus procidence**	20	6.64	6	1.99	20	6.6
**Jugular bulb procidence**	6	1.99	0	0	6	1.9
**Carotid artery procidence**	0	0	0	0	0	0
**Eustachian tube outlet obstruction**	75	24.9	10	3.32	88	29.2

*CT, computed tomography; COM, chronic otitis media*.

Concerning lesions' localizations, the involvement of tympanic and/or mastoid cavities was mentioned in about half of the CT reports, generally involving the tympanum more than the mastoid (*N* = 132 vs. *N* = 122); a more accurate depiction of the involvement of single subsites (such as epitympanum, mesotympanum, hypotympanus, aditus ad antrum, and/or antrum) was provided in a minority of cases; at the same time, information concerning the pattern of mastoid pneumatisation for surgical planning was never provided. Conversely, surgical data proved an almost constant involvement of the epitympanic region (*N* = 287), followed by mesotympanum (*N* = 78) and hypotympanum (*N* = 16); mastoid involvement was also found to be very common (*N* = 174), with frequent extension from the aditus to antrum (*N* = 156).

Concerning the ossicular chain, on the CT reports signs of erosion were mentioned in 233 cases, slightly overestimating subsequent surgical findings that confirmed bony erosion in 222 cases. In the majority of reports, it was described a global involvement of ossicular chain without specifying individual ossicles; indeed, ossicles were radiologically described only in 21 cases, with the malleus eroded in 21 patients and incus in 12 patients (whereas the stapes and oval window were never mentioned). Conversely, the post-surgical description depicted malleus erosion in 90 cases, incus erosion in 172 cases, and stapes erosion in 131 cases.

The involvement of tegmen tympani was overestimated on CT reports as described in 72 patients compared to 32 cases confirmed at surgery; the discrepancy concerned partial erosion patterns (*N* = 60 vs. *N* = 16 at surgery), whereas every case of complete interruption of tegmen tympani described on the CT report was then confirmed at surgery (*N* = 12). Korner's septum and scutum were only sporadically mentioned when eroded, while no data concerning sigmoid plate erosion was ever provided.

Similarly to partial tegmen tympani erosion, also bony labyrinth erosion was overestimated on CT reports (*N* = 39 vs. *N* = 17 at surgery), and in a large majority of cases an accurate description of single substructures was not provided. Only in 12 reports labyrinthine substructures involvement was depicted, with nine cases of incomplete fistula and three cases of complete fistula reported; however, CT reports slightly underestimated labyrinth involvement, with surgical findings highlighting the presence of incomplete fistula in 13 patients and complete fistula in 4 patients.

As well as for the above-mentioned structures, also the facial nerve canal involvement was underestimated on the CT reports (*N* = 18 cases vs. *N* = 76 cases at surgery); also in this case, as for the ossicular chain, no information regarding the most involved trait of the facial canal was provided. Similarly, also the patency state of the Eustachian tube was almost never mentioned on CT reports and described as obstructed in only 10 patients (compared to *N* = 75 at surgery).

Finally, anatomical variants of the intra-temporal venous sinuses are almost never described on CT reports, with the only exception of the sigmoid sinus procidence in six patients (vs. *N* = 20 at surgical exploration); the position of jugular bulb was never described, despite significant procidence detected in six patients at surgery. Similarly, no data concerning the vertical and the horizontal intra-temporal segments of internal carotid artery was provided, although no case of procidence/dehiscence was then documented at surgery.

When globally testing the strength of the agreement between surgical reports and CT reports, the inter-rater reliability was higher when comparing with structured CT checklist (*k* = 0.68 - substantial agreement) than with unstructured CT reports (*k* = 0.40 - fair agreement).

## Discussion

The role of HRCT in preoperative assessment of chronic inflammatory middle ear pathology is still debated because of its limited discriminatory power in distinguishing between cholesteatoma and non-cholesteatomatous inflammatory tissue, especially in early disease when bony erosion or remodeling is not present (11–14). However, HRCT of the petrous temporal bone is still considered the reference method for the accurate assessment of disease extension, identification of anatomic variants, and pre-surgical planning based on multiplanar reconstruction (15). However, beside a standardized volumetric acquisition CT protocol, it is also important to provide a structured radiologic report including a comprehensive depiction of all the osseous landmarks within the temporal bone. Standardized reporting is particularly helpful in defining best surgical access and identifying eventual anatomical variants that may increase the risk of life-threatening intra-operative complications (16–19). With this knowledge in this retrospective study, we revised unstructured preoperative CT reports of external non-tertiary diagnostic centers of patients who underwent middle ear surgery at our institute for chronic hyperplastic and/or cholesteatomatous otitis media, critically analyzing quantitative and qualitative data provided by the radiologist compared to the ones obtained on the same dataset by using a structured CT checklist (Supplementary Materials).

### Nature of Middle Ear Disease

Due to the limited ability of unenhanced CT scan in distinguishing among cholesteatoma, abscess, and granulation tissue based on densitometric values alone, limited data concerning hypothesis about the nature of acquired middle ear inflammatory lesion is generally provided in the majority of revised radiological reports. However, some information can be inferred from the simultaneous presence of opacification in typical location, bone erosion, and tissue remodeling (20).

The literature data on the CT sensitivity and specificity in determining lesion nature and origin differ among studies, with results ranging between 50 and 60% (11, 13) and 80 and 95% (12, 21, 22). However, this limitation has been largely overcome thanks to the inclusion of DW-MRI in the diagnostic flow chart to assess the presence of admixed cholesteatomatous tissue (2–5). Nowadays, the presence of cholesteatoma > 3 mm can be easily revealed by low values on apparent diffusion coefficient (ADC) maps derived from DW-MRI sequences, both for primary diagnosis and in cases of residual/recurrent disease (2–5, 23).

### Lesion Location

The presence of pathological tissue affecting the middle ear and the mastoid can be detected at CT examination both on axial and coronal planes (12, 13, 24–26), and its extension can be easily defined with multiplanar reconstructions. However, as further confirmed by our analysis, a structured report is crucial to ensure an accurate depiction of all anatomical landmarks necessary for a successful surgical planning (16–19).

### Ossicular Chain

In our experience, an ossicular chain description is generally provided in CT reports, with a good correspondence between radiological (77%) and surgical findings (74%). However, the most of these descriptions concerned the ossicular chain in its entirety, with limited information regarding single ossicles. As in the English literature, the malleus and the body of incus were easily recognized in CT images both in coronal and in axial scans and accurately described, while the long process of the incus and the stapes were more difficult to dissociate and not always included in the reporting (11, 12, 24, 27–30).

### Tegmen Tympani

**Figure 2:**
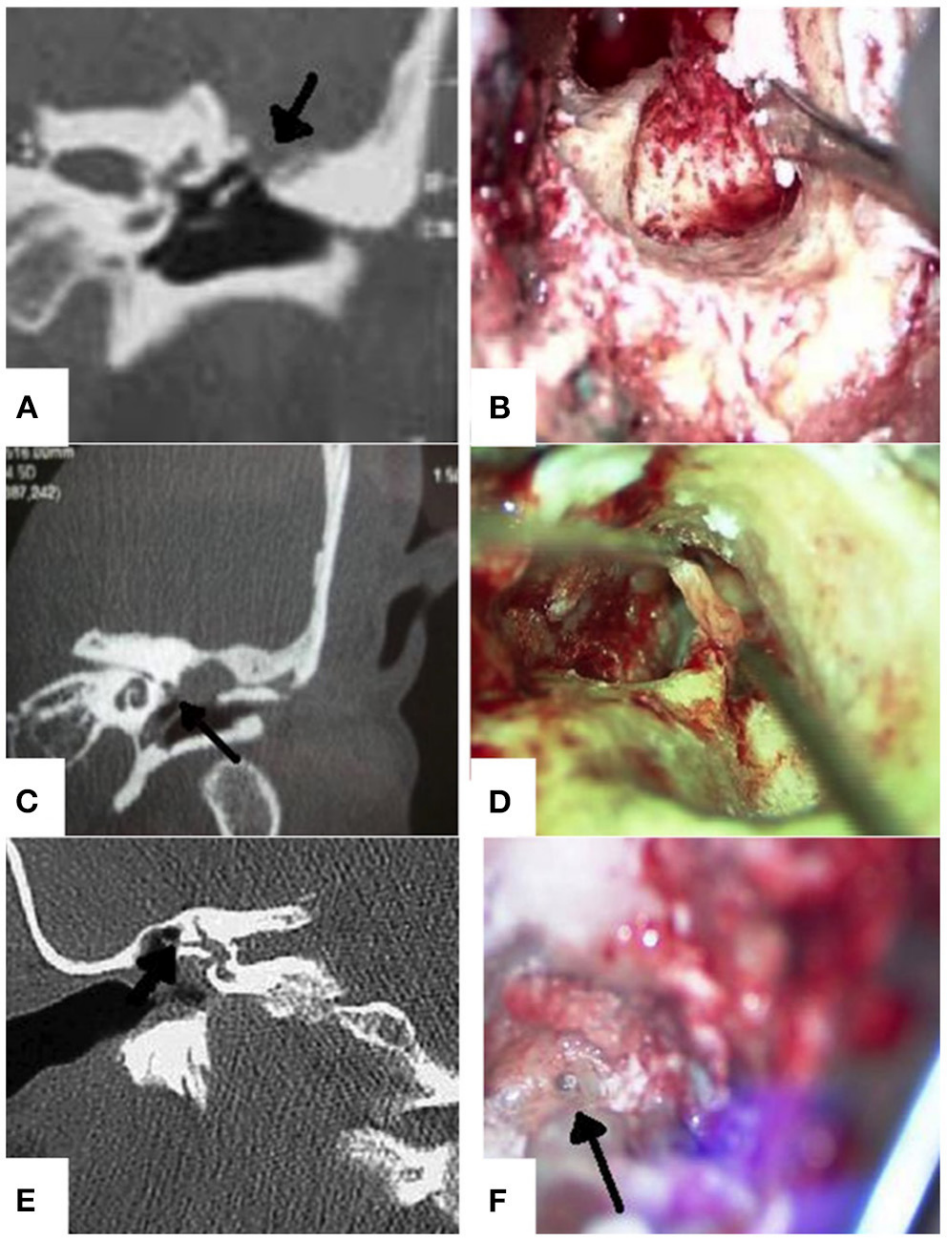
**(A)** Left ear - Tegmen tympani interruption on CT scan, coronal view (black arrow); **(B)** Left ear - tegmen tympani interruption on surgical field under microscopic view; **(C)** Left ear - Eroded tympanic tract of facial nerve on CT scan, coronal view (black arrow); **(D)** Left ear - cholesteatomatous matrix detachment from eroded tympanic tract area on surgical field under microscopic view; **(E)** Right ear - LSC wall fistula on CT scan, coronal view (black arrow); **(F)** Right ear - LSC wall fistula on surgical field under microscopic view (black arrow).

In our study, the prevalence of erosion and/or focal interruption of tegmen tympani (Figures 2A,B) was higher in CT reports (24%) than in surgical findings (11%) (11, 12, 24–28). This tendency to overestimation is probably due to old generation scan systems used for acquisition, more prone to “partial volume” effects and frequently inducing to overestimate erosion, especially on coronal planes evaluation.

### Facial Canal and Bony Labyrinth (Lateral Semi-Circular Canal)

Despite it is well-known that the presence cholesteatoma-mediated erosion can be accurately diagnosed by a HRCT scan (16), the description of smaller or more complex anatomical structures such as facial canal (Figures 2C,D) or LSC (Figures 2E,F) was generally underestimated in CT reports. Indeed, as reported in literature, focal erosion of the thin bone wall of facial canal is not always easy to identify, especially when lined by inflammatory tissue (11, 12, 24–28). The description is more accurate in case of canal dehiscence (typically in the tympanic tract), or when unilateral involvement is present allowing for comparison with contralateral ear (24, 31, 32). Similarly, to tegmen tympani and facial canal, also for bony labyrinth the presence of hypodense tissue wrapping and covering bony walls, can lead to overestimating the erosion (12, 24–28). Moreover, labyrinth fistulas have been classified in three subgroups: type I, defined as an erosion of the bony labyrinth with intact endosteum; type II, defined as a complete bony fistula with opened perilymphatic space; and type III, defined as the above-mentioned type II with concomitant involvement of the membranous labyrinth. Type I accounts for incomplete fistulas, whereas types II and III account for complete fistulas; as shown in different literature reports, the identification of complete fistulas involving LSC is generally easier compared to incomplete and very small fistulas (16, 31–33).

### Additional Findings: Vascular Variants, Eustachian Tube Patency, and Mastoid Pneumatisation

The pre-operative knowledge of possible procidence of jugular bulb/sigmoid sinus and internal carotid artery canal, as well as information about Eustachian tube patency are important for a successful surgical planning, both for optimizing the technique choice and adopting cautions in surgical dissection. Unfortunately, these data were often insufficient or even absent, being reported in a minority of cases; moreover, no formal statement concerning the absence of anatomical variants was never included in negative cases. Similar considerations also apply to petrous apex, mastoid, and infra-labyrinthine pneumatisation patterns (34).

We can therefore assume that the variability across radiological reports should be attributed at least in part to radiologists' expertise and experience in ENT imaging, as well as high volumes of diagnostic examinations referring to non-specialized centers. In this light, the presented analysis could have been at least in part influenced by some uncontrollable sources of variability (i.e., radiologists' experiences in ENT imaging, CT technical aspects, scanner used for CT acquisition, etc.), as it usually happens in daily clinical settings. Structured reporting has been identified as a possible long-term solution to this discrepancy, ensuring clarity, immediacy, and consistency in communication, without limiting radiologists' abilities to communicate advices and opinions (35), in order to optimize surgical planning and post-surgical monitoring (34, 36–40). In this light, the proposed structured checklist to apply to chronic inflammatory middle ear pathology can optimize the communication with surgeons, also reducing the risk of inter-observer variability at diagnosis and during follow-up examinations. Many authors previously suggested the utility of a shared model for staging of middle ear inflammatory pathology based on CT findings (16–19); however, at present, no unified consensus was reached. This structured and comprehensive checklist aims to summarize previous models, including an extensive evaluation of anatomic structures along with major anatomical variants of relevance for surgery, providing a final overview that could be indicative of disease severity and local aggressiveness (Supplementary Materials).

## Conclusion

Although MRI examination is crucial to determine the nature of chronic inflammatory pathology of the middle ear, CT scan is still mandatory to evaluate temporal bony structures, optimize pre-surgical planning and manage intra-operative complications.

To obtain all the necessary information concerning lesions nature, extension, and involvement of adjacent anatomical structures, standardization of the radiology report structure is strongly recommended. Standardized reporting ensures completeness and simplifies the identification of key information, reducing the risk of ambiguity and imaging data misinterpretation. This is even more important for high-complexity anatomical regions, such as petrous temporal bone, where detailed description of pathology-related findings and possible morphostructural variants are crucial to guiding the surgeon in pre-treatment planning and extensive disease removal, also minimizing treatment-related complications and contributing to preserve normal functions.

Therefore, we propose a possible radiological report model to apply to chronic inflammatory middle ear pathology, integrating previous literature evidences, and providing a checklist based on imaging findings. This standardized report is functional for improving communication between surgeon and radiologist and for overcoming possible imaging limitations. However, to improve these data, comparison with other tomographic techniques (such as Flat-Panel Volume CT and Cone-Beam CT) and possible inclusion of different MRI criteria based on DW techniques should be further analyzed.

## Data Availability Statement

The raw data supporting the conclusions of this article will be made available by the authors, without undue reservation.

## Ethics Statement

The studies involving human participants were reviewed and approved by Comitato Etico C. Romano - Università degli Studi di Napoli Federico II. The patients/participants provided their written informed consent to participate in this study.

## Author Contributions

MC, AD, and CR: conception and design of the study. AD, MM, EC, and GD: data acquisition, analysis, and interpretation. MC and AD: drafting. GM and, AE: final approval. MC, GM, and AE: agreement to be accountable for all aspects of the work in ensuring that questions related to the accuracy or integrity of any part of the work are appropriately investigated and resolved. All authors contributed to the study conception and design according to ICMJE recommendations.

## Conflict of Interest

The authors declare that the research was conducted in the absence of any commercial or financial relationships that could be construed as a potential conflict of interest.
