# Supplementary material for: Computed-Tomography-Structured Reporting in Middle Ear Opacification: Surgical Results and Clinical Considerations From a Large Retrospective Analysis
Source: Front Neurol. 2021 Feb 24;12:615356. doi: 10.3389/fneur.2021.615356 (PMC7943916; doi:10.3389/fneur.2021.615356)
Supplement: Supplementary file 1 [file Table_1.docx]

**Supplementary Table 1**

Structured radiological checklist for chronic inflammatory middle ear pathology.

| **Radiological features based on HRCT** | | | **Presence** | **Absence** |
| --- | --- | --- | --- | --- |
| **Opacification location and extension** | | |  |  |
|  | Tympanum: | Epitympanum | ☐ | ☐ |
|  |  | Mesotympanum | ☐ | ☐ |
|  |  | Hypotympanum | ☐ | ☐ |
|  | Mastoid: | Mastoid Antrum | ☐ | ☐ |
|  |  | Mastoid Air Cells | ☐ | ☐ |
| **Ossicular chain involvement** | | |  |  |
|  | Displacement without erosion | | ☐ | ☐ |
|  | Malleus erosion | | ☐ | ☐ |
|  | Incus erosion | | ☐ | ☐ |
|  | Stapes erosion | | ☐ | ☐ |
|  | Oval window erosion | | ☐ | ☐ |
| **Facial nerve canal involvement** | | |  |  |
|  | Intact | | ☐ | ☐ |
|  | Dehiscent | | ☐ | ☐ |
|  | Eroded: | Proximal tympanic segment | ☐ | ☐ |
|  |  | Distal tympanic segment | ☐ | ☐ |
|  |  | First genu | ☐ | ☐ |
|  |  | Mastoid segment | ☐ | ☐ |
| **Labyrinth involvement with semicircular canal erosion* (according to Dornhoffer and Milewski)** | | | | |
|  | Intact | | ☐ | ☐ |
|  | Incomplete fistula | | ☐ | ☐ |
|  | Complete fistula | | ☐ | ☐ |
| **Middle ear bony wall erosion** | | |  |  |
|  | Tegmen tympani: | Intact | ☐ | ☐ |
|  |  | Thinned | ☐ | ☐ |
|  |  | Eroded | ☐ | ☐ |
|  | Scutum: | Intact | ☐ | ☐ |
|  |  | Blunted | ☐ | ☐ |
|  |  | Eroded | ☐ | ☐ |
|  | Korner’s septum: | Intact | ☐ | ☐ |
|  |  | Eroded | ☐ | ☐ |
|  | Sigmoid plate: | Intact | ☐ | ☐ |
|  |  | Eroded | ☐ | ☐ |
|  | Mastoid cortex: | Intact | ☐ | ☐ |
|  |  | Eroded | ☐ | ☐ |
| **Eustachian tube patency** | | |  |  |
|  | Patent | | ☐ | ☐ |
|  | Partially obstructed | | ☐ | ☐ |
|  | Completely obstructed | | ☐ | ☐ |
| **Intra/Extra cranial complications** | | |  |  |
|  | Extra-cranial extension | | ☐ | ☐ |
|  | Intra-cranial extension | | ☐ | ☐ |
| **ADDITIONAL: Vascular anatomical variants** | | |  |  |
|  | Jugular bulb: | Normal positioning | ☐ | ☐ |
|  |  | Procidence | ☐ | ☐ |
|  |  | Dehiscence | ☐ | ☐ |
|  | Internal Carotid Artery: | Normal positioning | ☐ | ☐ |
|  |  | Procidence | ☐ | ☐ |
|  |  | Dehiscence | ☐ | ☐ |
| **ADDITIONAL: Pattern of mastoid pneumatisation** (according to Dexian Tan et al.)** | | | | |
|  | Petrous apex pneumatisation: | Hypopneumatisation | ☐ | ☐ |
|  |  | Mild pneumatization | ☐ | ☐ |
|  |  | Moderate pneumatization | ☐ | ☐ |
|  |  | Hyperpneumatisation | ☐ | ☐ |
|  | Mastoid pneumatisation: | Hypopneumatisation | ☐ | ☐ |
|  |  | Mild pneumatization | ☐ | ☐ |
|  |  | Moderate pneumatization | ☐ | ☐ |
|  |  | Hyperpneumatisation | ☐ | ☐ |
|  | Infralabyrinthine pneumatisation: | Non-pneumatised | ☐ | ☐ |
|  |  | Partly pneumatised | ☐ | ☐ |
|  |  | Well-pneumatised | ☐ | ☐ |
| **ADDITIONAL: Tissue characterization based on DW-MRI** | | | **(Computed ADC value)** | |
|  | Abscess | |  | |
|  | Cholesteatoma | |  | |
|  | Granulation tissue | |  | |

***Legend:***

** Labyrinth involvement with semicircular canal erosion (according to Dornhoffer and Milewski)*

*Intact: no bony erosion*

*Incomplete fistula: erosion of the bony labyrinth with intact endosteum (type I)*

*Complete fistula: complete erosion with opened perilymphatic space, without (type II) or with (type III) concomitant involvement of the membranous labyrinth.*

*** Pattern of mastoid pneumatisation (according to Dexian Tan et al.)*

*Petrous apex pneumatisation:*

*Hypopneumatisation - No air cells are present in the vicinity of the inner ear*

*Mild pneumatisation - Less than half of the petrous apex medial to the labyrinth is pneumatized*

*Moderate pneumatisation - More than half of the petrous apex medial to the labyrinth is pneumatized*

*Hyperpneumatisation - Most of the petrous apex area medial to the labyrinth is composed of air cells*

*Mastoid pneumatisation:*

*Hypopneumatisation – Pneumatization anteromedial to the line drawn at the most anterior point of the sigmoid sinus*

*Mild pneumatisation - Pneumatization up to the space between the 2 arbitrary lines drawn at the most anterior point of the sigmoid sinus and the most lateral aspect of the sigmoid sinus*

*Moderate pneumatisation - Pneumatization up to the space between the 2 arbitrary lines drawn between the most lateral aspect of the sigmoid sinus and the most posterior point of the sigmoid sinus*

*Hyperpneumatisation – Pneumatization beyond the arbitrary line drawn at the most posterior point of the sigmoid sinus*

*Infralabyrinthine pneumatisation:*

*Non-pneumatised - absence of air cells at the most inferior and medial portion of the petrous apex*

*Partly pneumatised - limited pneumatized bone below the labyrinth*

*Well-pneumatised - well-pneumatized temporal bone at the most inferior and medial portion of the petrous apex below the internal auditory meatus on the sagittal plane*
